# Patient and physician perspectives on engaging in palliative and healthcare trials: a qualitative descriptive study

**DOI:** 10.1186/s12904-021-00856-6

**Published:** 2021-10-14

**Authors:** Valeria Cardenas, Anna Rahman, Jenna Giulioni, Alexis Coulourides Kogan, Susan Enguidanos

**Affiliations:** 1 Leonard Davis School of Gerontology, University of Southern California, 3715 McClintock Ave., GER 208B, Los Angeles, CA 90089 USA; 2 Division of Biokinesiology and Physical Therapy, University of Southern California, Los Angeles, CA 90033 USA; 3 Keck School of Medicine, Department of Family Medicine and Geriatrics, University of Southern California , Alhambra, CA 91803 USA

**Keywords:** Palliative care, Qualitative study, Referral, Primary care physicians, Caregivers, Recruitment

## Abstract

**Background:**

Researchers are encountering increasing challenges in recruiting participants for palliative and healthcare research. This paper aims to understand challenges to and methods for engaging physicians and seriously ill patients and their caregivers in research studies.

**Methods:**

Between October 2019 to July 2020, we conducted qualitative interviews with 25 patients, proxies, and caregivers participants who were eligible for a randomized controlled trial of home-based palliative care and 31 physicians from participating accountable care organizations. Using thematic analysis, we analyzed participants’ responses to identify concepts and key ideas within the text. From these initial concepts, core themes around barriers to research and preferred research recruitment approaches were generated.

**Results:**

Themes from patient and caregiver interviews included time constraints, privacy concerns, lack of research familiarity, disconnect with research institution, self-perceived health status, and concerns with study randomization. Physician-identified barriers focused on time constraints and study randomization. Patient and caregiver recommendations for study recruitment included in-person recruitment, recruitment at healthcare providers’ offices, recruitment via mail, additional study information, and frequent calls. Physician recommendations were related to placement of flyers at clinics, financial incentives, and formal events.

**Conclusions:**

Findings demonstrated that although patients and caregivers prefer that their physicians recruit them for health-related research studies, physicians identified time constraints as a consistent barrier to research involvement.

**Supplementary Information:**

The online version contains supplementary material available at 10.1186/s12904-021-00856-6.

## Background

Healthcare research is critical to improving treatment, reducing disease and symptoms, and reducing avoidable costs of health care [1], yet studies have found increasing difficulty in engaging participants in research trials [2]. Additionally, recruitment and retention are often the most challenging aspect of conducting research [3–5], let alone when patients with serious illness are targeted [6, 7]. This is concerning given that the need for improved care among seriously ill patient has been well documented [8, 9] and remains a focus of research efforts nationally [10].

Palliative care research, with its focus on improving quality of life for patients with serious illness, faces significant challenges in appropriate patient identification and enrollment in research trials. For example, investigators of an early Palliative Care Research Cooperative trial who conducted interviews with study site personnel identified three primary challenges to recruitment related to patients and physicians: (1) locating trial-eligible patients; (2) severity of patient illness; and (3) physician/caregiver gatekeeping over patients [11]. Additionally, insufficient patient recruitment also has been identified as a challenge in the majority of National Cancer Institute funded studies of palliative care [12]; further highlighting the importance of understanding challenges and facilitators to patient recruitment.

Involving physicians in research also has been identified as a challenge [13–15]. Although primary care physicians (PCPs) report positive experiences in conducting research [16], PCPs face financial and time constraints in their work, preventing them from engaging in research [17]. Additionally, healthcare providers may fear burdening their patients and may lack clear understanding of the research topic or what will be asked of participants, thereby serving as gatekeepers to research participation among their patients [18]. This gatekeeping behavior has made it difficult for researchers to recruit seriously ill patients into studies, including palliative care trials [19–21].

In a wide-reaching qualitative synthesis review of facilitators and barriers related to patient and caregiver involvement in palliative care research, Chambers and colleagues identified several themes that ranged from definition of research and palliative care to organization cultural and diversity [21]. Notably researchers identified a paucity of rigorous studies on patient and caregiver barriers and facilitators to palliative care research and called for more rigorous investigations and studies that included patients and caregivers in the design and implementation.

Furthermore, despite more than a decade of research on palliative care practices, there remain significant gaps in research to advance this field. Researchers analyzed more than 600 published studies on palliative care to identify priorities for future research. Among the most frequently identified areas in need of more research were the perspectives and needs of patients, relatives, and providers [22].

While previous studies have documented barriers encountered in palliative care research, few have studied these barriers from patient, caregiver, and physician perspectives who had been approached to participate in a randomized controlled trial (RCT). Additionally, few studies focus on identifying strategies to improve research trial engagement among physicians and seriously ill patients and their family members. To fill this gap, we conducted a qualitative descriptive study to identify challenges to engaging patients, proxies, caregivers, and physicians in research and their preferred strategies methods for research recruitment. We also aimed to understand the relationship between the patient and physician perspectives and elicit specific recommendations to better engage both patients and physicians in palliative care research, a unique contribution of this investigation.

## Methods

To achieve this aim, we conducted individual interviews with primary care physicians, patients, and caregivers who previously had been approached to be part of a RCT [23]. The original RCT aimed to compare the effectiveness of home-based palliative care and primary care enhanced by added training in palliative care principles among seriously ill patients with congestive heart failure, chronic obstructive pulmonary disease, or advanced cancer and their caregivers [23], but was terminated due to under enrollment [24]. The RCT and this qualitative descriptive study were individually approved by the Institutional Review Board of the University of Southern California. Our study report follows the Consolidated Criteria for Reporting Qualitative Studies (COREQ) guidelines [25].

### Recruitment

From October 2019 to July 2020, we used purposive sampling to recruit PCPs from two accountable care organizations (ACOs) and patients (and their caregivers) who had been previously approached to participate in an RCT. We purposively selected both those who had agreed to be part of the research study as well as those who declined. Patients and caregivers were initially mailed a letter explaining the current study and invited to participate in a telephone interview. We followed the letter with a telephone call. When a patient lacked capacity to consent, the research assistant interviewed the proxy. We used various strategies to recruit PCPs for the current study; PCPs were invited to participate through faxed and mailed study flyers, telephone calls to their office, and holiday cards. In a couple of cases, we visited their office to invite them to participate. All participants were invited to participate in a 30-min interview.

### Interview protocol

The study team, comprised of experts in palliative care and research, developed a semi-structured protocol to elicit information about PCPs, patients’, and caregivers’ perceptions on research participation, including their perceived barriers to participation and their suggestions for overcoming these challenges. The interview protocol was reviewed by patient and caregivers study advisors as well as by an outside researcher familiar with our study. After the first interview, the study protocol was slightly modified for ease of administration.

The protocol included questions inquiring about patient, proxy, and caregiver concern with research participation and their preferred method of study recruitment. PCPs were asked what they perceived as barriers to providing patient referrals to research studies and suggestions for overcoming these barriers. See Additional file 1 for interview questions. Patients, proxies, and caregivers received a $50 (USD) gift card for their participation. Physicians initially received a $150 (USD) gift card, which was later increased to $300 (USD) to improve participation rates. Patient, proxy, and caregiver interviews were conducted by a female study manager with a Master of Public Health degree and PCP interviews by a doctoral trained female (A.C.K.) with more than 10 years of research experience, including qualitative interviewing. The interviewers disclosed their role In the research study to study participants. All interviews were conducted via telephone with the exception of one PCP interview taking place in-person. All interviews were audio-recorded, transcribed verbatim, and lasted between 10 and 54 min.

At the conclusion of each interview, demographic information was collected from all participants. Patient, proxy, and caregiver information included age, gender, ethnicity, marital status, education, work status, and current medical conditions. PCPs reported their age, gender, ethnicity, country of birth, and number of years working in healthcare and in their current position.

### Analysis

Interview transcripts were transferred to an Excel sheet by trained research assistants and were analyzed by three researchers (V.C., J.G., Y.Z.). Using thematic analysis [26], researchers familiarized themselves with the data by reading the transcripts and writing down initial concepts and key ideas. The team convened to discuss this initial list of ideas and consolidated related concepts before arriving at a single list of themes. The same researchers reread and independently coded the transcripts using this list of themes, while comparing and linking themes throughout the process. The researchers met again and final codes were compared and discussed until 100% agreement was reached between coders. In the final step, researchers extracted examples for each theme. We conducted this process separately for both the PCP interviews and the patient/proxy/caregiver interviews. Thematic saturation was reached after the 10th interview for patients/proxy/caregivers and after the 14th interview for PCPs, however we continued to code all transcripts. Final themes were shared with our study investigator team and our patient and caregiver stakeholders for feedback. Stakeholder feedback did not change any of the final themes.

## Results

From October 2019 to July 2020, we interviewed 56 participants: 17 patients, eight caregivers/proxies, and 31 PCPs. Of the patients, proxies, and caregivers, we recruited 15 from among 109 RCT-eligible participants who refused to participate in the earlier study and 10 from the 28 participants who enrolled in the RCT. Among potential patients and caregivers, five had died, five were unreachable, three refused to participate, and three declined for other reasons. Of the PCPs, we contacted 198 physicians of which 31 responded to our invitation to participate in our study.

### Participant demographic characteristics

Table 1 reports the demographic characteristics of the participating patients, caregivers, proxies, and PCPs. Among the 17 patient participants, the majority were female (70.6%), white (82.4%), and married (70.6%). More than half (53%) had attained a college degree or higher, and 64.7% were retired. Patients reported currently having one or more medical conditions. Six patients (35.3%) had enrolled in the larger RCT, while 11 (64.7%) had declined to participate.

**Table 1:** Participant Characteristics (*N* = 56)

	All	Patients Only	Caregivers/Proxy Only	Physicians Only
	(N = 56)	(*N* = 17)	(*N* = 8)	(*N* = 31)
Characteristics	n (%)			
Age (mean ± SD)	58.91 ± 10.74	64.06 ± 9.83	55.88 ± 15.23	56.87 ± 9.17
Gender				
Male	26 (46.4%)	5 (29.4%)	4 (50%)	17 (54.8%)
Female	30 (53.6%)	12 (70.6%)	4 (50%)	14 (45.2%)
Ethnicity				
Black/African American	2 (3.6%)	1 (5.9%)	1 (12.5%)	0 (0%)
White/Caucasian	32 (57.1%)	14 (82.4%)	4 (50%)	14 (45.2%)
Hispanic/Latino	4 (7.1%)	1 (5.9%)	1 (12.5%)	2 (6.5%)
Asian	11 (19.6%)	1 (5.9%)	1 (12.5%)	9 (29%)
Other	7 (12.5%)	0 (0%)	1 (12.5%)	6 (19.4%)
US Born^b^				
Yes	18 (58.1%)			18 (58.1%)
No	13 (41.9%)			13 (41.9%)
Marital Status^a^				
Single	3 (12%)	2 (11.8%)	1 (12.5%)	
Married/Living with a partner	16 (64%)	12 (70.6%)	4 (50%)	
Widowed	2 (8%)	0 (0%)	2 (25%)	
Divorced	4 (16%)	3 (17.7%)	1 (12.5%)	
Education^a^				
High School Graduate	3 (12%)	2 (11.8%)	1 (12.5%)	
Some College	9 (36%)	6 (35.3%)	3 (37.5%)	
College Graduate	11 (44%)	7 (41.2%)	4 (50%)	
Post Graduate	2 (8%)	2 (11.8%)	0 (0%)	
Work Status				
Work full-time	6 (24%)	3 (17.7%)	3 (37.5%)	
Unemployed	4 (16%)	3 (17.7%)	1 (12.5%)	
Retired	15 (60%)	11 (64.7%)	4 (50%)	
Years working in health care^b^				
11 to 15	5 (16.1%)			5 (16.1%)
16 to 20	2 (6.5%)			2 (6.5%)
Over to 20 years	24 (77.4%)			24 (77.4%)
Years working in certain position^b^				
Less than a year	0 (0%)			0 (0%)
1 to 5	4 (12.9%)			4 (12.9%)
6 to 10	4 (12.9%)			4 (12.9%)
11 to 15	4 (12.9%)			4 (12.9%)
16 to 20	7 (22.6%)			7 (22.6%)
Over to 20 years	12 (38.7%)			12 (38.7%)
Medical Condition^a^				
Cancer	5 (20%)	4 (23.5%)	1 (12.5%)	
COPD	4 (16%)	4 (23.5%)	0 (0%)	
Heart Disease	8 (32%)	8 (47.1%)	0 (0%)	
Liver Disease	2 (8%)	2 (11.8%)	0 (0%)	
Diabetes	8 (32%)	8 (47.1%)	0 (0%)	
Arthritis	9 (36%)	7 (41.2%)	2 (25%)	
Study Status				
Enrolled	17 (30.4%)	6 (35.3%)	4 (50%)	7 (22.6%)
Declined	39 (69.6%)	11 (64.7%)	4 (50%)	24 (77.4%)

Footnotes:

^a^ Patients & Caregivers (*N* = 25)

^b^ Physicians Only (N = 31)

Among the eight caregivers and proxies, 50% were male, white, married, college graduates, and retired. Caregivers/proxies reported having fewer medical conditions than patients. Half the caregivers/proxies had a family member who had enrolled in the larger RCT and the other half had declined to participate. All proxies interviewed were family members, with one identifying as a caregiver as well. Additional participant demographics are reported in Table 1.

Of the 31 physician participants, 17 (54.8%) were male and 14 (45.2%) were female. Nearly half (45.2%) were white, 9 (29%) were Asian, six (19.4%) identified as other, and two (6.5%) were Latino/Hispanic. Ages ranged from 39 to 75 years (M = 57; SD = 9.17). More than half (58.1%) of the physicians were foreign-born. Most (77.4%) reported working in healthcare for more than 20 years.

### Themes

From the data, we identified six themes related to barriers to healthcare research and eight recommendations for study recruitment. Themes are presented below and in Table 2.

**Table 2:** Themes Identified

CATEGORY/THEME	DEFINITION	EXAMPLE COMMENT	FREQUENCY OF THEME (%)	
Perceived Physician/Patient/Caregiver Barriers			Physician	Patient/Proxy/Caregiver
**Time**	Time constraints due to physicians’ high workload and patient’s and caregiver’s care	“I’ve been so busy. I’ve been going through so much trials and tribulations, I haven’t been able to, to respond to the mail, the information.” (PT 23)	87%	44%
**Privacy Concerns**	Concerns with how researcher had obtained their information and how their personal information is shared	“When they reached out to me personally, I was more worried about HIPPA. I was worried that any information I provided was going to go into third party hands.” (CG 25)	0%	48%
**Research Familiarity and Attitudes**	When approached, primary care physicians were unaware of the current study and patients and caregivers ambivalent views of research	“Well, I think I was a little taken back at first, ...because I had never been in a study or any kind of research.” (PT 2)	0%	28%
**Disconnect with Research Institution**	Patient’s and caregiver’s lack of connection with the research institution	“When I saw [name of research institution] it wasn’t part of the group I was with. And I’ve never been there... I was never treated at [research institution], so I was kind of wondering how they would be. How would I get involved with them? It’s not a name I’m used to around this area. You know, we have [local institution], or we have [local institution], or we have [local institution].” (PT 6)	0%	20%
**Randomization**	Participants expressed concerns with the randomization aspect of a randomized control trial	“If they really needed the palliative care and they’re getting usual supportive care. I think, they are being short changed…either you get the better care or not.” (PCP 2)	26%	16%
**Patient’s Health Condition**	Patients felt unable to participate due to the severity of their current diagnosis	“I just had six months out of my… third heart operation and I just didn’t really know what you were doing, so I just didn’t want to get involved.” (PT 11)	0%	22%
**Recommendations**				
***Recruitment Approaches***				
**In-person Recruitment**	Researchers should recruit participants in person	“In person, I believe it’s better because you can look [at a] person and explain better than [mail or email]. You don’t see anybody … [in] email or mail. I believe in person [is] better.” (PT 21)	0%	25%
**Recruitment at Healthcare provider’s office**	Researchers should provide information and recruit patients at healthcare provider’s office	“So at least if it’s coming from somewhere along my healthcare chain, then at least to me, I might pay half attention to it, you know what I mean?” (PT 17)	0%	28%
**Flyer at Clinic**	There should be a brochure with study information for patient, caregivers, and physicians at healthcare provider’s office	“It could be helpful to bring brochures, having out some handout… that they can read more details, I think it is a good idea.” (PCP 47)	23%	0%
**Letter Recruitment**	Patient’s and caregiver’s request to receive a letter as initial source of recruitment	“Maybe start out with a letter in the mail, followed by a phone call explaining how the program works, and what it’s going to lead to, and who’s going to be involved.” (PT 10)	0%	22%
**Outreach Frequency**	Patients and caregivers recommended having multiple points of contact during recruitment	“…doing a lot of check-ins or saying the same information in a couple different ways. And then also laying out like, this is step one, what would happen, step two, what would happen.” (PT 22)	0%	16%
**More Information**	Researchers should provide an outline and better explanation with all study details	“It should be explained better as to who they are, who everyone represents better, so that you understand that better.” (PT 7)	0%	19%
***Incentives***				
**Money**	Physicians recommended researchers offer a financial incentive for their time	“For me, again, pay me enough money, I’ll do it.” (PCP 4)	84%	0%
**Dinner/Formal Event**	Researchers should host a formal event to explain research study to physicians	“Take us out to meet some of the people, small trip to the university, out to dinner.”(PCP 33)	29%	0%

### Barriers to healthcare research

#### Time

Patients, proxies, caregivers, and PCPs identified time constraints as a barrier to research participation. Patients, proxies, and caregivers shared that their time was constrained by taking care of their health or the health of their loved one.

“I felt like there was so much going on and I was concerned about adding another something I need to do.” (PT 22)

Nearly all PCPs mentioned being unable or unwilling to participate in research due to their busy schedules. One physician (PCP 1) stated that:

“The main challenge… is that when you’re in the middle of a really busy clinical day, it’s really hard to have one extra thing that you need to do.”

#### Privacy concerns

Several patients, proxies, and caregivers mentioned that they were wary about how the research team had obtained their information and whether their information would be provided to a third party. One patient (PT 17) shared her concerns about potential fraud and the legitimacy of the recruitment call. The lack of notification of the study prior to the call contributed to her distrust and concern about a potential telephone scam:

“I don’t know why I was being contacted, it was basically just someone calling me out of the blue, and it could have been just something fake, [someone] trying to get my personal information.”

#### Disconnect with the research institution

Also contributing to the distrust in the research recruitment call was that patients stated they had no connection with the research institution and questioned how a non-local institution would conduct research in their town. One patient (PT 7) described the geographic distance between the research institute and his residence as a source of disconnect:

“You guys are out in [location of research institution], I was in [hometown], so I don’t know how you would have had somebody in my [hometown].

#### Research familiarity and attitudes

Patients, proxies, and caregivers shared their negative perceptions about research and the novelty of participating in a research study. Some mentioned how the word “research” made them uncomfortable, not wanting to enroll because they had no prior experience with research or simply disliked interviews. Some patients had discussed the study with their doctors, and their doctor who had no knowledge of the research study.

“To be honest, I’ve never been in one [research study], it is probably something that I’m not comfortable in… I don’t [see] myself involved in those kinds of things.” (PT 6)

#### Randomization

PCPs, patients, proxies, and caregivers were concerned about the randomization process and the potential to be assigned to a group that did not receive palliative care. Similarly, several physicians expressed concern that patients might not receive needed services due to randomization.

“It becomes a little concerning…in my mind, they need the [service] and if [the patient] is going to get it or not, becomes more like an ethical thing.” (PCP 61)

A caregiver enrolled in the previous RCT said her only concern with the study would have been “not being chosen for the in-home care.” (CG 2).

#### Patients’ health condition

Many patients said they could not participate in the earlier RCT because they were too sick. Patients discussed having gone through multiple hospitalizations, being too ill, and not being able to ‘deal with it all.’ As one patient (PT 7) said:

“When you’re really sick, it’s something hard to just concentrate on answering random multiple-choice questions…because you’re not really sure where your own health is going at the time.”

### Recommendations

#### Recruitment approaches

##### In-person recruitment

Many participants recommended that research recruitment occur in-person. Some believed in-person recruitment could facilitate participant understanding of the study and help researchers understand patients’ current health status. One caregiver (CG 13) said:

“…there is nothing better than to do [research recruitment] personally, no telephone, no filling out brochures… It is much easier to meet with a person for 30 minutes to an hour.”

##### Recruitment at healthcare Provider’s office

More specifically, several patients, proxies, and caregivers recommended that recruitment occur at the physician’s office or prior to being discharged from the hospital. As one caregiver mentioned (CG 15):

“I’m thinking when [the patient] has a doctor’s appointment—if you would be able to go to a doctor’s appointment and while they were waiting for their doctor’s appointment [they could be approached to participate in a study] …or before they left the hospital.”

##### Flyers at clinics

PCPs suggested that researchers leave flyers for patients at their clinics and an outline of the research project for physicians. A physician (PCP 43) proposed:

“If I have an outline of the research projects that you are conducting that will help me basically, keep your [study in] mind for when I see the patients.”

##### Letter recruitment

Some participants recommended that they receive study information by mail before receiving the first recruitment phone call. A patient (PT 8) said:

“A letter in the mail is way better than just a phone call because most people won’t answer the phone because they don’t know the [phone] number.”

##### Outreach Frequency

Several patients, proxies, and caregivers recommended the research team contact them multiple times to make sure the message comes across appropriately. One patient emphasized the importance of multiple recruitment calls:

“…making that second and third call back… to let them know what you are doing. And eventually they’re going to see what you’re trying to tell them. (PT 6)

##### More information

Many patients, proxies, and caregivers said they needed a clearer explanation of the study and of palliative care; they recommended that the research team provide multiple sources of information. An enrolled participant from the previous RCT (#10) suggested:

“[having]…an outline of how it's going to be conducted and what it's going to lead to: ‘We will be calling you every month to ask questions,’ and ‘We will be referring you to other local organizations for one-on-one help.’”

#### Incentives

##### Money

While financial incentives were not mentioned by patients, proxies, and caregivers*,* nearly all PCPs said a financial incentive would encourage physician research participation. One physician (PCP 2) said he would need “… enough of a reimbursement to make it worth my while.”

##### Formal event

Several PCPs suggested that the research teams host a formal event to explain their study. A physician (PCP 47) mentioned:

“… offering dinners at nice restaurants and then saying these opportunities we’d like to get you engaged [in]...”

## Discussion

This study identified challenges related to conducting and participating in research as perceived by PCPs, patients, and caregivers/proxies. Through this new understanding of the relationship between the patient, proxy, caregiver, and physician perspectives, this study provides a better understanding of how researchers could work with physicians to build participant trust in research by providing a “warm handoff” during recruitment. Additionally, our findings highlight the need for researchers to address physician’s challenges, namely in ensuring adequate time and/or incentives are provided for physician inclusion in research trials.

In particular, nearly all PCPs in this study identified time as a major challenge in participating in research, a finding widely supported by previous research [13, 16, 17, 21, 27]. Studies have shown that despite physician awareness of potential long-term benefits of participating in research, healthcare providers are inundated with the immediate demands of patient care; thus, patient referrals to research studies become a lower priority [14].

Patients, proxies, and caregivers felt their time was constrained by their poor health and their medical care needs. In a multi-site RCT of patients with end-stage renal disease, nearly half (47%) had refused participation due to the severity of their disease [28]. Similarly, a palliative care clinical trial found the most commonly cited reason for refusing study participation was that the patient felt too sick [29].

Participants also identified challenges that were specific to the RCT’s design. Several patients, proxies, and caregivers were concerned about randomization and not being able to select the palliative care intervention themselves. Often patients view randomization as a loss of control and prefer that their doctor select their treatment [30, 31]. Those who had no previous research involvement were wary of what a research study would entail. With this lack of research familiarity came privacy concerns: participants were nervous about how their information was obtained and with whom it would be shared.

Patients also were concerned about their physician’s lack of awareness of the RCT. Lack of awareness of ongoing clinical trials among physicians was identified as a major challenge to patient recruitment in five academic medical centers in the United States (U.S.) [32]. PCPs’ lack of awareness and their attitudes towards research can influence patient exposure to research participation.

A couple of the barriers we identified related to issues of trust in relation to the organization conducting study outreach. From the patient, proxy, and caregiver perspective, the research institute did not have a clear connection with their healthcare provider, which may have heightened concerns around privacy. Additionally, “cold calling” without prior notification of study recruitment (via letter, through their physician, etc) also heightened their sense of distrust in the legitimacy of the research call.

### Recruitment approaches

Patients, proxies, and caregivers suggested multiple strategies and approaches to recruiting participants for research studies. These included recruitment via mail, in-person, or at healthcare providers’ offices. These different approaches have all demonstrated some success. Face-to-face recruitment yielded the highest response rates in a study of three health networks in the U.S. [33]. Others have suggested that physician involvement and recruitment at their office is the most important and effective recruitment strategy [34, 35]. Physician recommendations and referrals to research studies have been a strong factor to participation [36]. While our participants preferred their PCP to introduce the study, PCPs in our study discussed the many time constraints that would prevent them from actively recruiting patients for research. Instead, they suggested recruitment efforts that would not impinge on their time, such as placement of fliers in their clinic.

In addition, patients, proxies, and caregivers suggested sending a recruitment letter prior to making the first phone call, which has been an integral part of study recruitment [37]. When receiving the first call, participants requested numerous phone calls to grab their attention, while others disputed this approach earlier.

#### Incentives

Nearly all PCPs discussed how a financial incentive could encourage them to participate in research. Lack of financial incentives has been identified as a barrier to participating in research with PCPs [16]. Studies have found that some physicians believe monetary compensation demonstrates that the physician’s time is valued, [27] and that this incentive also can increase a physician’s likelihood to participate in research [38].

Interestingly, incentives did not arise in the interviews conducted among patients, proxies, and caregivers. Anecdotally, just one respondent discussed financial incentives and mentioned that healthcare was more important than a monetary incentive. While a systematic literature review on health research study participation found that provision of monetary incentives increased patient participation between 4 to 23% [39], little is known about the usefulness of monetary incentives in palliative care research or research among seriously ill patients.

### Strengths and limitations

To our knowledge, this is the first study to investigate barriers and recommendations to overcoming these barriers from the perspective of PCPs, patients, proxies, and caregivers after the conclusion of a previous research trial. This study was conducted with a purposive sample of RCT-eligible patients, caregivers, and physicians contracted with an ACO in the U.S. Responses may have been influenced by their experiences with the larger RCT’s recruitment efforts and their level of participation in the RCT. The participants interviewed in this study may have declined participation in the larger RCT but were willing to take part in this qualitative descriptive study. Therefore, the results do not represent the perspective of potential participants who decline all research participation, another strength of this study in that perspectives include those consenting and declining a larger research trial. Additionally, this study focuses exclusively on stakeholder perceptions of research recruitment, and does not include strategies for reducing participant attrition, another challenge when conducting research with populations with serious illness [40, 41].

Future studies are needed to compare effectiveness of recruitment strategies to determine best practices for engaging physicians and seriously ill patients in research.

## Conclusion

This study found that patients, proxies, and caregivers may be cautious when approached for palliative care research participation, and therefore prefer in-person recruitment for research trials as well as being recruited by their physician. However, we also found that physicians have limited time to participate in studies. These findings underscore the importance of offering incentives as a strategy to increase physician research involvement.

## Supplementary Information

**Additional file 1.**

## Data Availability

The datasets used and analyzed during the current study are available from the corresponding author on reasonable request.

## Abbreviations

PCP: Primary care physician
RCT: Randomized controlled trial
COREQ: Consolidated Criteria for Reporting Qualitative Studies
ACOs: Accountable care organizations
PT: Patient
U.S.: United States
