# Supplementary material for: Patient and physician perspectives on engaging in palliative and healthcare trials: a qualitative descriptive study
Source: BMC Palliat Care. 2021 Oct 14;20:158. doi: 10.1186/s12904-021-00856-6 (PMC8515687; doi:10.1186/s12904-021-00856-6)
Supplement: Supplementary file 1 — Additional file 1. [file 12904_2021_856_MOESM1_ESM.docx]

**APPENDIX A**

***Patient/Caregiver Interview Protocol***

1. Thinking back to (INSERT DATE) when our research team member first called you to talk to you about our palliative care study, what was the reason you (accepted/declined) to participate?
   1. Could we have done something differently to change your mind?
   2. Did you have any concerns about the services that were part of study participation?
2. For the next two questions, think about the information that was provided to you over the phone
   1. What information did you dislike?
   2. What information did you like or was helpful?
3. Please explain any concerns you may have had when our research team reached out to you.
4. Thinking about the conversation that you had with our research team member about the study, how can we improve that conversation?
5. Tell me about any concerns you have about participating in research studies in general.
6. When researchers need to recruit patients with complicated illness for a study what in your opinion, is the best way to approach them?
   1. Are there any approaches that you believe will not work or should be avoided?

***Physician Interview Protocol***

1. I want to start by asking you about challenges PCPs face in engaging in research projects.  From your perspective, what are your thoughts about being involved in a research study that relates to primary care or your primary care patients?
   1. Specify, what about a study that involves identifying and referring patients?
   2. Taking additional training?
2. What could researchers do to better support or motivate PCPs to be part of research projects?
   1. What incentives could promote participation in such trials?
3. What recommendations do PCPs have for engaging PCPs in these studies?
4. What concerns did you have about your patients participating in this study?
5. What concerns, if any, did you have related to home-based palliative care?
6. Were there other reasons you did not want your patients involved in the study?
